# Acupuncture for perimenopausal insomnia: a systematic review and meta-analysis

**DOI:** 10.3389/fmed.2025.1673994

**Published:** 2025-10-13

**Authors:** Ruqin Yang, Shumin Zhang, Gaoyangzi Huang, Xin Tang, Qifu Li, Ya Huang, Yuanzheng Deng, Hongyang Wang, Ziwen Chen, Taipin Guo, Fanrong Liang

**Affiliations:** ^1^School of Second Clinical Medicine, Yunnan University of Chinese Medicine, Kunming, China; ^2^College of Acupuncture and Tuina, Chengdu University of Traditional Chinese Medicine, Chengdu, Sichuan, China

**Keywords:** perimenopausal insomnia, acupuncture, randomized controlled trials, systematic review, meta-analysis

## Abstract

**Objective:**

Perimenopausal insomnia (PMI) significantly compromises the quality of life and the physical and mental wellbeing of perimenopausal women. Although many randomized controlled trials (RCTs) indicate that acupuncture alleviates PMI symptoms, comprehensive evidence on its efficacy and safety is lacking. We conducted a meta-analysis to systematically evaluate the therapeutic efficacy and safety of acupuncture for PMI, aiming to provide robust, evidence-based guidance for clinical practice.

**Methods:**

A total of 12 trials were included in the study, involving 499 participants in the acupuncture group and 495 in the control group, all of which provided data for the meta-analysis. Quality assessment was performed using the Cochrane Risk of Bias (ROB) tool. RevMan version 5.4 and Stata 18.0 software were used for meta-analysis and sensitivity analysis.

**Results:**

A total of 10 studies were included, with the random-effects model showing that acupuncture significantly improved sleep quality compared to the drug control group, as evidenced by a reduction in Pittsburgh Sleep Quality Index (PSQI) scores [mean difference (MD) = −2.26, 95% confidence interval (CI) (−4.23 to −0.29), *p* < 0.00001]. Subgroup analyses revealed that acupuncture outperformed Estazolam (MD = −2.89, *p* < 0.00001). However, it did not outperform alprazolam (MD = −2.30, *p* = 0.27) or diazepam (MD = −6.55, *p* < 0.00001). Compared to sham acupuncture, acupuncture resulted in a significantly greater reduction in PSQI scores (MD = −4.85, *p* = 0.0001). Acupuncture also led to reductions in luteinizing hormone (LH) (MD = −6.55, *p* < 0.00001) and follicle-stimulating hormone (FSH) (MD = −12.12, *p* = 0.002), and an increase in estradiol (E2) levels (MD = 11.96, *p* < 0.00001). One study reported a significant improvement in Insomnia Severity Index (ISI) scores (MD = −3.64, *p* = 0.0003). Adverse event data were reported in only 4 of the 12 studies.

**Conclusion:**

Current evidence of low to moderate quality suggests that acupuncture may offer potential benefits and demonstrate good safety for PMI. However, these findings require further validation through large-scale, multicenter, double-blind RCTs using standardized protocols.

**Systematic review registration:**

https://www.crd.york.ac.uk/PROSPERO/, Identifier (CRD42018092917).

## Introduction

Perimenopausal insomnia (PMI) is a persistent sleep disorder that occurs in women around the time of menopause, usually characterized by difficulty falling asleep, light sleep, frequent nighttime awakenings, early morning awakenings, decreased sleep quality, and daytime dysfunction (1). Epidemiologic surveys revealed that premenopausal women experience sleep disorders in 16–42% of cases, while perimenopausal women experience sleep disorders in up to 75–81% of cases (2, 3). In the United States, the prevalence of perimenopausal sleep disorders in women is 31–42% (4). PMI seriously affects the physical and mental health of patients. Long-term sleep disorders may lead to problems such as fatigue, poor concentration, anxiety, and depression, and increase the risk of developing cardiovascular disease, diabetes, and autonomic nervous system disorders, significantly affecting patients’ quality of life, social functioning, and increasing the economic burden on society (5, 6).

Currently, PMI is mainly treated with drugs and hormone replacement therapy. However, these treatments often result in adverse effects, such as drug dependence, high recurrence rates, and increased risks of cardiovascular diseases and cancer, causing some patients to reject these therapies (7–11). Consequently, non-pharmacological therapies, such as acupuncture, cognitive therapy, tai chi, and yoga, have gained popularity. Acupuncture is preferred by many patients due to its safety and absence of side effects (12, 13). A previous comprehensive analysis, which reviewed more than 30 clinical trials, confirmed the efficacy of acupuncture in relieving PMI symptoms and recommended acupuncture as a treatment option for PMI (14).

Given the increasing use of acupuncture for PMI, numerous randomized controlled trials (RCTs) have explored its impact on sleep quality, yet results remain inconsistent. This underscores the need for systematic reviews and meta-analyses to evaluate the overall evidence. Previous meta-analyses suggested that acupuncture may offer a safe alternative or adjunct to pharmacological treatments, with some improvements in follicle-stimulating hormone (FSH) and estradiol (E2) (15, 16). However, these studies focused mainly on comparisons with Western medications, excluding other control interventions, and did not incorporate recent RCTs. This study aims to provide a comprehensive review, including recent trials, and perform detailed subgroup analyses to rigorously assess acupuncture’s efficacy and safety relative to other treatments for PMI.

## Methods

This study was registered with the International Prospective Register of Systematic Reviews (PROSPERO) (registration number: CRD42018092917) and was conducted in accordance with the Preferred Reporting Items for Systematic Reviews and Meta-Analyses (PRISMA) guidelines (17). The detailed PRISMA checklist is provided in Supplementary Table S1. All acupuncture-related technical details (e.g., needle specification, depth of insertion, needle retention time, and treatment course) were extracted in accordance with the Standards for Reporting Interventions in Clinical Trials of Acupuncture guidelines (STRICTA); further details are provided in Supplementary Table S2.

### Literature search and selection

A systematic search was conducted across nine databases, including PubMed, Embase, Cochrane Library, Web of Science, China National Knowledge Infrastructure (CNKI), Wanfang Data, China Science and Technology Journal Database (VIP), China Biomedical Literature Database (CBM), and the Chinese Clinical Trial Registry (ChiCTR), from their inception up to 31 December 2023. Only studies published in English or Chinese were included. Our search strategy had three main parts: (1) Perimenopausal insomnia, menopausal insomnia, menopausal sleep disorders, and perimenopausal sleep disorders; (2) Acupuncture, needling, manual acupuncture, electroacupuncture, floating acupuncture, and acupoint burrowing; (3) Type of study: RCTs. The complete and detailed search strategies for all databases, developed in accordance with PRISMA 2020 guidelines, are provided in Supplementary Table S3.

### Eligible criteria

Studies that meet the following criteria were included according to the population, intervention, comparison, results, and study design (PICOS) frame:

P (participants): Perimenopausal women diagnosed with primary insomnia. Trials lacking specific diagnostic criteria were excluded.

I (interventions): The experimental group was treated with acupuncture treatments, including hand acupuncture, auricular acupuncture, floating acupuncture, electroacupuncture, cupping therapy, and moxibustion.

C (comparison): The control groups did not receive any treatment or received treatment other than acupuncture, such as sham acupuncture, conventional medication, psychosocial interventions, or other conventional interventions. The before and after itself as a control group, or herbal medicine as a control group, were excluded.

O (outcomes): This study evaluated the efficacy and safety of acupuncture in treating PMI. The Pittsburgh Sleep Quality Index (PSQI) served as the primary outcome measure. Secondary outcomes included serum levels of FSH, luteinizing hormone (LH), and E2, the Insomnia Severity Index (ISI) total score, and adverse events.

S (study Design): RCTs.

### Exclusion criteria

This study included RCTs or controlled clinical trials of acupuncture for PMI, some of which employed blinding or allocation concealment. The following studies were excluded: (1) duplicate publications; (2) reviews, systematic reviews, meta-analyses, animal studies, case reports, commentaries, conference abstracts, research protocols, master’s theses, doctoral dissertations, bulletins, and consensus statements; (3) articles with titles or interventions that did not fit; (4) articles without primary outcome measures; (5) studies for which the full text or underlying data were unavailable.

### Literature screening and data extraction

All studies extracted from the nine databases were imported into EndNote software for initial screening. Two independent researchers (RY and SZ) screened the titles and abstracts based on the inclusion and exclusion criteria. They then reviewed the full-text articles for secondary screening. Data extraction was also performed independently by the two researchers (RY and SZ), and the results were exchanged for comparison. Any disagreements were resolved through discussion with a third researcher (GH). Data extraction included study characteristics and outcome indicators. The study characteristics included the first author’s name, year of publication, study design, sample size, age, type of acupuncture, acupoint selection, a detailed description of the control group, adverse events, and the type of reported outcomes. Measured outcomes included PSQI, LH, FSH, E2, and ISI scores after the intervention. Finally, if the data were incomplete, attempts were made to contact the authors for the missing data.

### Bias risk assessment

Two researchers (RY and SZ) independently assessed the risk of bias for each included study, with disagreements in the assessment being resolved through negotiation with a third reviewer (GH). The risk of bias assessment consisted of 6 parts: (1) randomized sequence generation; (2) allocation concealment, blinding of subjects and staff; (3) blinding of outcome assessment; (4) incomplete outcome data; (5) selective reporting; and (6) other biases. We considered funding sources and baseline imbalance as other potential sources of bias. Each component was rated as having a low, unclear, or high risk of bias. Studies were of low overall quality if more than half of the evaluation entries were rated as high risk (18).

### Statistical analysis

The study data were analyzed using meta-analysis software RevMan version 5.4 and Stata 18.0 software. For continuous variables, analyses were performed using mean difference (MD) or standardized mean difference (SMD). When different instruments were used to measure outcome indicators, the SMD with a 95% confidence interval (CI) was applied. In contrast, the MD with 95% CI was used when the same instruments were employed. Furthermore, *p* < 0.05 was considered statistically significant. Inter-study heterogeneity was evaluated using the chi-square test and the I^2^ statistic. The results indicated that heterogeneity was negligible when the I^2^ statistic ranged from 0 to 50%, moderate when it ranged from 50 to 75%, and high when it ranged from 75 to 100%. The random-effects model was primarily used to pool the data, as it provides a more conservative estimate in the presence of clinical heterogeneity. The fixed-effects model was considered only if no significant statistical heterogeneity was observed (I^2^ ≤ 50% and *p* ≥ 0.10 in the *χ*^2^ test). When heterogeneity was present, subgroup analyses were conducted based on different control interventions, and sensitivity analyses were performed to explore the sources of heterogeneity. In this meta-analysis, publication bias was assessed both visually, using funnel plots, and statistically, with Egger’s linear regression test for outcomes involving more than 10 studies. To evaluate the robustness of the findings, sensitivity analyses were performed by sequentially excluding each study and by removing studies with a high risk of bias. Furthermore, we plan to use the trim-and-fill method to estimate the potential impact of publication bias on the pooled effect size.

## Results

**Figure 1:**
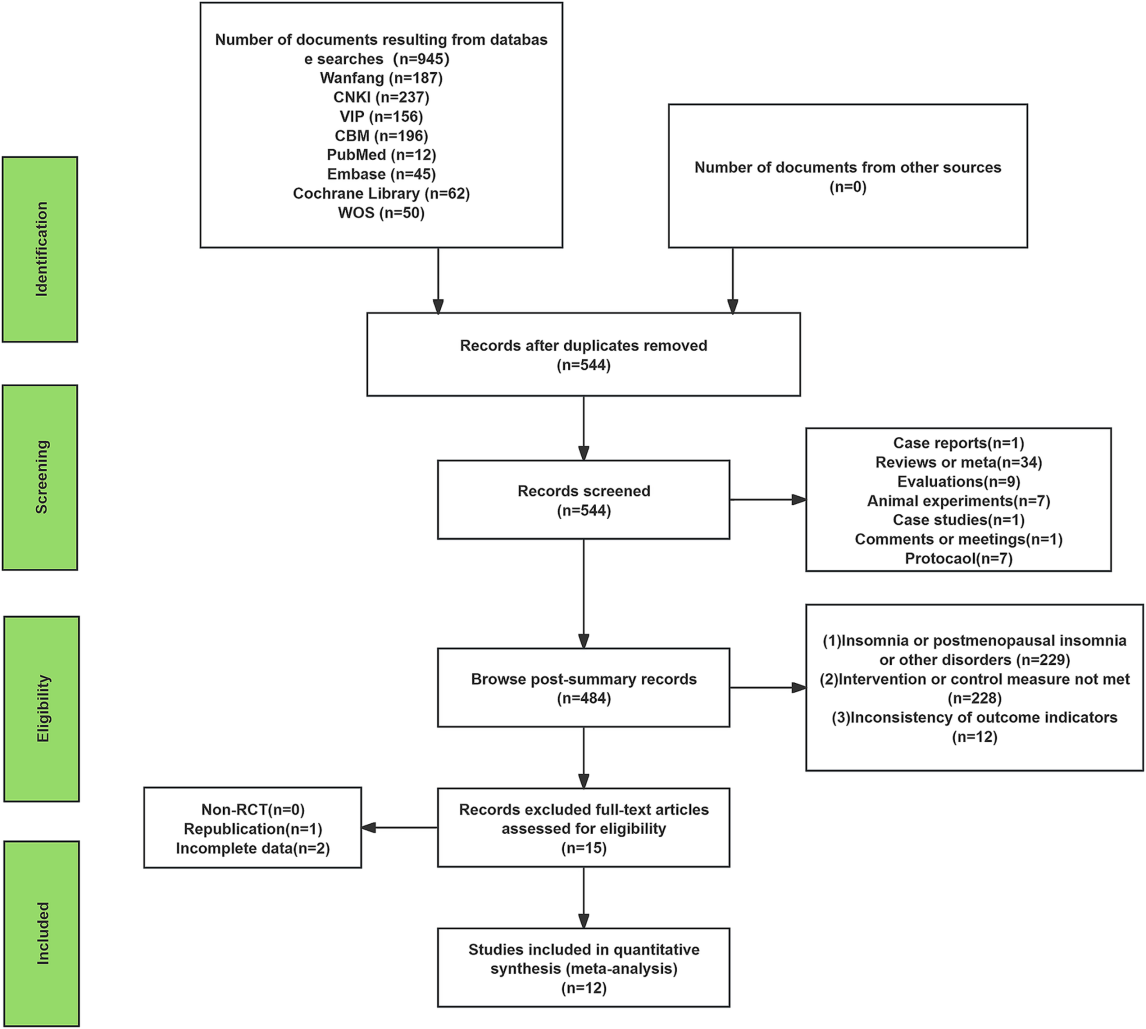
Flow diagram of study screening.

A total of 945 studies were retrieved by searching 9 databases, with 401 duplicates removed, leaving 544 studies. A total of 529 studies were deleted and excluded after reading the title and abstract because they did not meet the inclusion criteria. Three additional studies were excluded due to not fitting the article type or the data being unavailable. Finally, 12 studies were included in the systematic review and meta-analysis (Figure 1).

A total of 12 RCTs involving 994 female patients with PMI were included, with 499 patients in the acupuncture group and 495 in the control group. The sample size per group in each study ranged from 25 to 81 participants. All included studies were conducted in China. The most frequently used intervention in the acupuncture group was acupuncture alone (*n* = 8) (19–26), followed by acupuncture combined with cupping (27, 28), with one study using acupuncture combined with moxibustion (29), and one using auricular acupuncture combined with cupping (30). In the control group, seven studies used conventional medication (eszopiclone) (19–21, 23, 25, 26, 30), two studies used conventional medication (alprazolam) (22, 24), one study used conventional medication (Diazepam) (29), and the other two studies used sham acupuncture (27, 28). The mode of administration for all 10 conventional drug treatment groups was oral (19–26, 29, 30). PSQI scores were reported in 12 studies (19–30), 3 studies reported E2 scores (20, 22, 26), 4 studies reported FSH scores (20, 22, 23, 26), 3 studies reported LH scores (20, 22, 26), and ISI scores were reported in 1 study (28). Adverse events were documented in 4 studies (26–28, 30). The 12 studies included 45 acupoints. Cupping involved the governor meridian and bladder meridians, and the types of acupoints included meridian points, extra nerve points, ear points, and head points. Each acupoint was used 1–7 times in different combinations, with the most used being Baihui (DU20), Anmian, Shenmen (HT7), Sishencong (EX-HN1), Sanyinjiao (SP6), Zhongwan (RN12), and Guanyuan (RN4) (Table 1).

**Table 1:** Characteristics of studies.

Author year country	Sample size genders age	Interventions (acupoints)	Outcomes	Side effects
Wei et al. (2013) (25) China	T: 31, all female 50.45 ± 3.50 C: 31, all female 8.97 ± 2.88	T: acupuncture (GV20, EX-HN1, BL17, BL18, AnMian) C: drug (Estazolam)	PSQI	T: NR C: NR
Bo et al. (2017) (30) China	T: 49, all female 53.2 ± 3.16 C: 45, all female 53.0 ± 3.89	T: Ear Points +Walking Jars C: drug (Estazolam)	PSQI	T: NR C: Dizziness, headache, Nausea and anorexia, fatigue
Jinuo et al. (2017) (23) China	T: 81, all female 48.17 ± 4.12 C: 81, all female 49.45 ± 3.98	T: Head+body needles (PC6, HT7, ST36, KI3, ST40, RN12) C: drug (Estazolam)	PSQI	T: NR C: NR
Wei et al. (2017) (19) China	T: 35, all female 45–53 C: 35, all female 46–55	T: acupuncture (KI6, BL62, DU20, AnMian) C: drug (Estazolam)	PSQI	T: NR C: NR
Zhao et al. (2023) (26) China	T: 40, all female 49.85 ± 2.46 C: 40, all female 50.17 ± 2.71	T: acupuncture (DU20, EX-HN1, HT7, RN4, SP6, KI6, LR3, AnMian) C: drug (Estazolam)	PSQI, LH, FSH, E2	T: None C: None
Wang (2017) (32) china	T: 28, all female 50.28 ± 2.32 C: 28, all female 50.37 ± 2.31	T: acupuncture (HT7, DU20, LR3, KI3, PC6, DU24, SP6, BG13) C: drug (Estazolam)	PSQI	T: NR C: NR
Wenxiong et al. (2021) (20) China	T: 45, all female 50.8 ± 4.1 C: 45, all female 50.2 ± 4.3	T: acupuncture (RN12, RN10, RN6, RN4, ST24, ST26, KI17, KI13, EX-HN1, AnMian) C: drug (Estazolam)	PSQI, LH, FSH, E2	T: NR C: NR
Xiang (2020) (22) China	T: 56, all female 48.8 ± 5.8 C: 56, all female 49.2 ± 6.1	T: acupuncture (BL15, BL13, BL18, BL23, BL20, BL17, HT7) C: drug (Alprazolam)	PSQI, LH, E2	T: NR C: NR
Yang (2021) (24) China	T: 30, all female 50.08 ± 2.17 C: 30, all female 50.12 ± 2.26	T: acupuncture (DU20, DU24, BG13, RN4, RN12, ST25, KI12, EX-CA1, KI3, SP6, LR3, BL23, BL32) C: drug (Alprazolam)	PSQI	T: NR C: NR
Xue (2017) (29) China	T: 25, all female 50.6 ± 3.3 C: 25, all female	T: acupuncture (HT7, PC6, SP6, ST36, EX-HN1, RN12, DU20) C: drug (Diazepam tablet)	PSQI	T: NR C: NR
Li et al. (2020) (28) China	T: 42, all female 52.12 ± 4.19 C: 42, all female 53.07 ± 3.81	T: acupuncture (DU20, DU24, DU29, RN6, RN4, SP6, HT7, Anmian) C: sham acupuncture (DU20, DU24, DU29, RN6, RN4, SP6, HT7, Anmian)	PSQI, ISI	T: Bleeding, pain C: Pain
Fu et al. (2017) (27)	T: 38, all female 52.0 ± 5.3 C: 38, all female 52.5 ± 5.9	T: acupuncture (BL23, BL18, LR14, GB25) C: sham acupuncture (BL23, BL18, LR14, GB25)	PSQI	T: NR C: 4 cases of Worsening insomnia

NR, no report; T, treatment group; C, control group; PSQI, Pittsburgh sleep quality index; LH, Luteinizing hormone; FSH, Follicle-stimulating hormone; E2, Estradiol; SIS, insomnia severity index.

### Risk of bias

**Figure 2:**
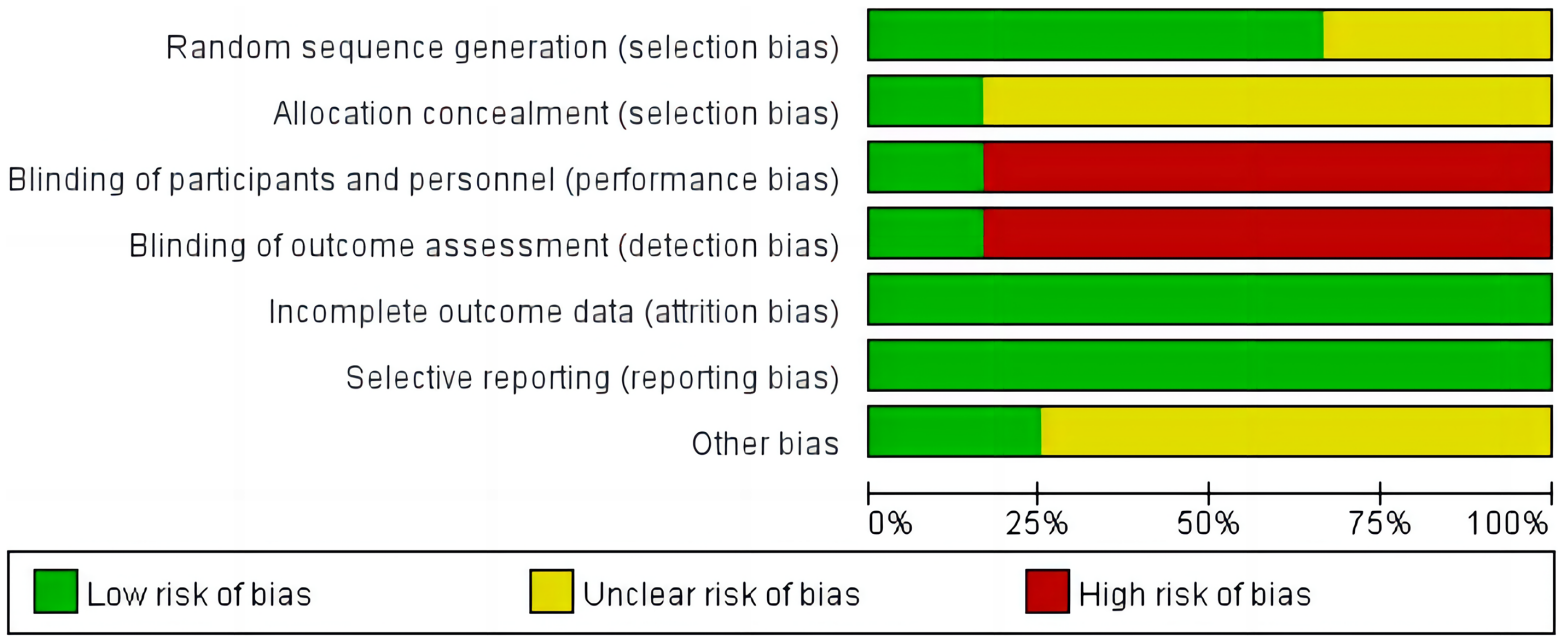
Risk of bias graph.

**Figure 3:**
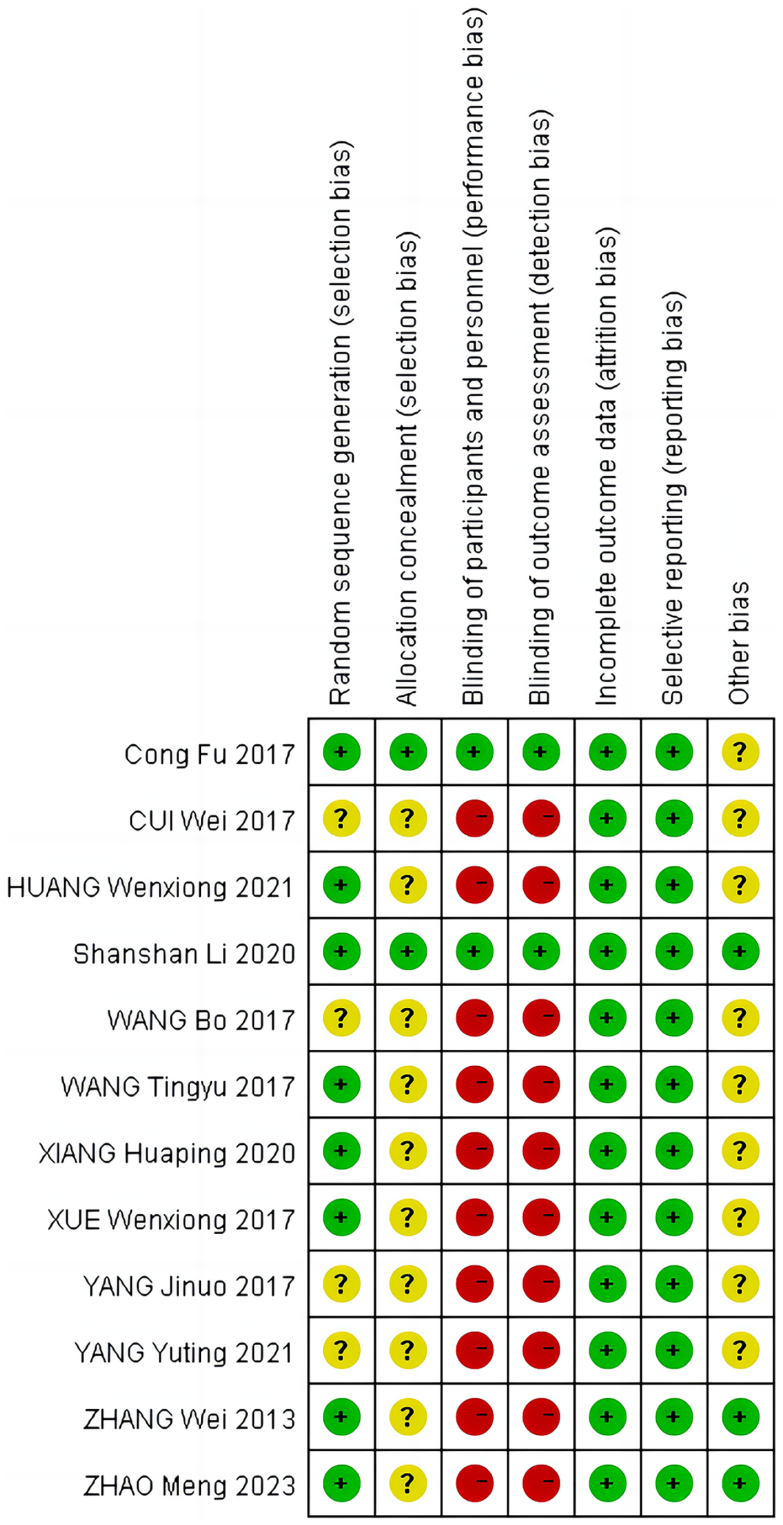
Risk of bias summary.

The 12 studies were assessed for risk of bias, with the simplified risk of bias shown in Figure 2, and the detailed risk of bias for each study shown in Figure 3. In terms of random sequence generation, eight studies were rated as low risk for using an appropriate random assignment method (random number table method) (20–22, 25–29). In comparison, four studies were rated as unclear for mentioning randomization but not describing the method in detail (19, 23, 24, 30). Regarding allocation concealment, two studies that described allocation concealment were rated as low risk (27, 28), while 10 studies that did not mention allocation concealment were rated as unclear (19–26, 29, 30). For subject blinding and outcome assessment, 2 studies were blinded to both subjects and outcome assessors and were rated as low risk (27, 28), while 10 studies did not mention blinding and were rated as high risk (19–26, 29, 30). Concerning incomplete outcome data, all 12 studies reported case dropouts and exclusion outcomes, with complete outcome data rated as low risk (19–30). Regarding selective reporting, all 12 studies reported complete outcome indicators and were rated as low risk (19–30). For other biases, three studies provided detailed information about the source of project funding. They were rated as low risk (25, 26, 28), while nine studies did not mention any source of funding and were rated as unclear (19–24, 27, 29, 30). More than half of each study’s entries were rated as low risk for the study to be considered high quality, and a total of 4 studies were qualified as high-quality research (25–28).

**Figure 4:**
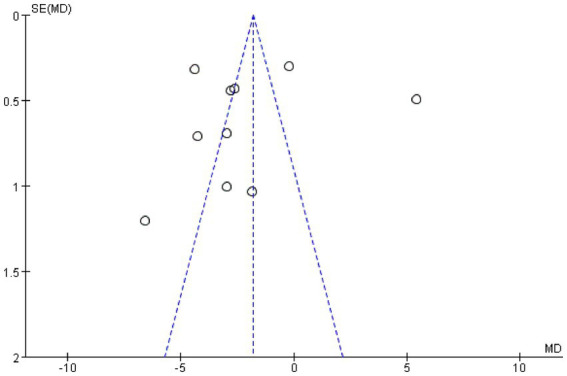
Funnel plot.

**Figure 5:**
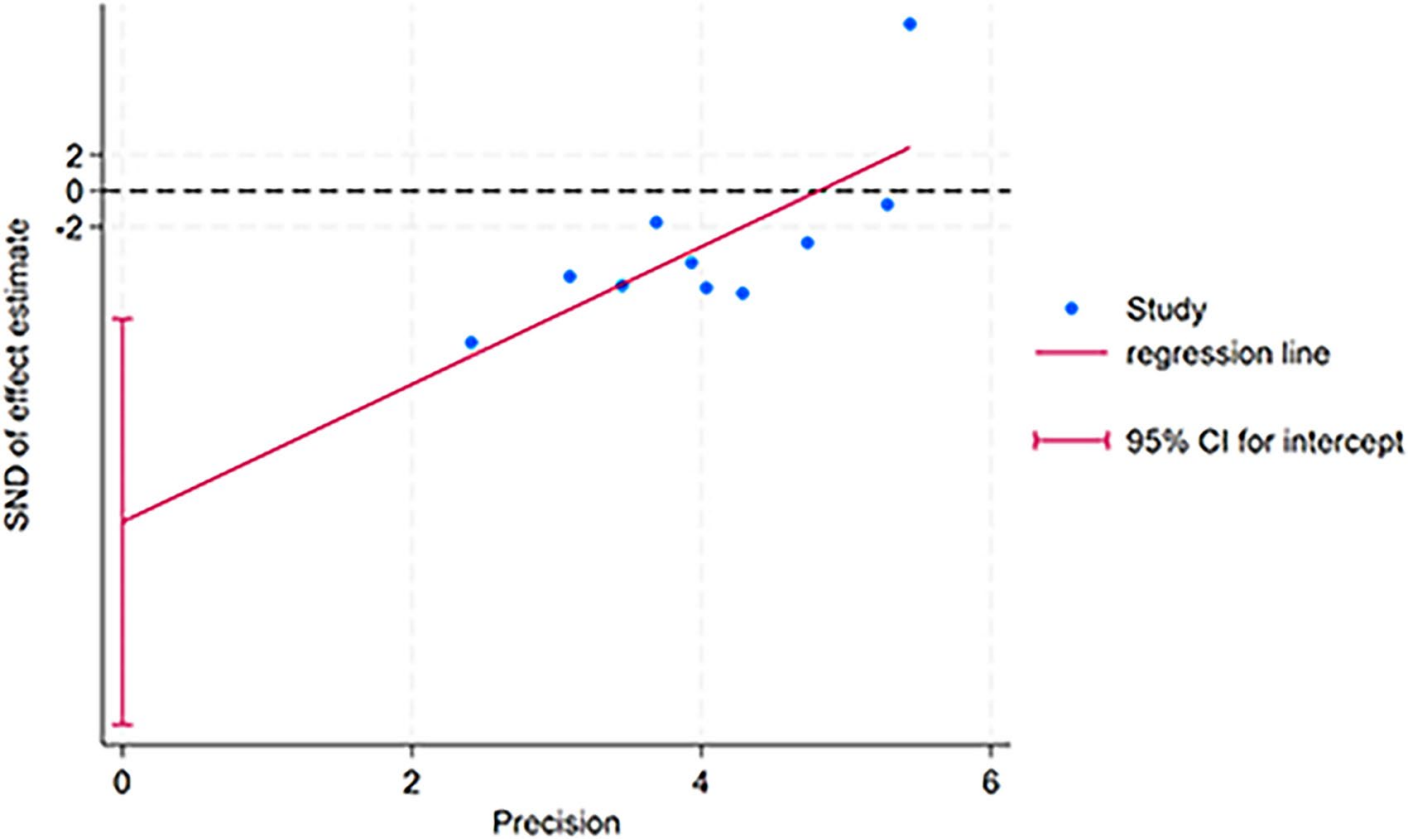
Scatter plot.

For the combined results of PSQI (acupuncture vs. Western medicine), 10 included studies were evaluated for publication bias. Visually, the funnel plot (Figure 4) displays significant asymmetry, with the effect sizes of small sample studies concentrated on the right side. It is underrepresented on the left, strongly suggesting the presence of bias or minor sample effects. Consequently, we performed Egger’s regression test on the 10 studies (acupuncture vs. Western medicine), and the results confirmed the significance of this asymmetry (intercept = −18.40, 95% CI: −29.68 to −7.12; *t* = −3.76, *p* = 0.006) (see Supplementary Figure S4). To evaluate publication bias, we plotted the Egger’s test scatterplot (Figure 5). Visually, the distribution of low-precision studies exhibited clear asymmetry, primarily around the regression line. This graphical indication suggests the presence of a small study effect, consistent with the quantitative results of Egger’s linear regression test (intercept = −18.40, *p* = 0.006). Sensitivity analysis showed that study 23 had a substantial impact on the summary results. After excluding this study, the Egger test for the remaining nine studies remained significant (*p* = 0.003), suggesting that the observed funnel plot asymmetry is not influenced by a single study, but may indicate persistent small study effects or publication bias based on the evidence (see Supplementary Figure S5). We further attempted to evaluate the impact of bias using the trim-and-fill method; however, due to algorithm instability in this dataset, the results from this method are not reported to maintain the accuracy of our findings. In summary, Egger’s test and funnel plots suggest that the combined results of this meta-analysis may be influenced by publication bias or small sample effects and should be interpreted with caution.

### Primary outcomes

#### PSQI

**Comparison between acupuncture and Western medicine.**

**Figure 6:**
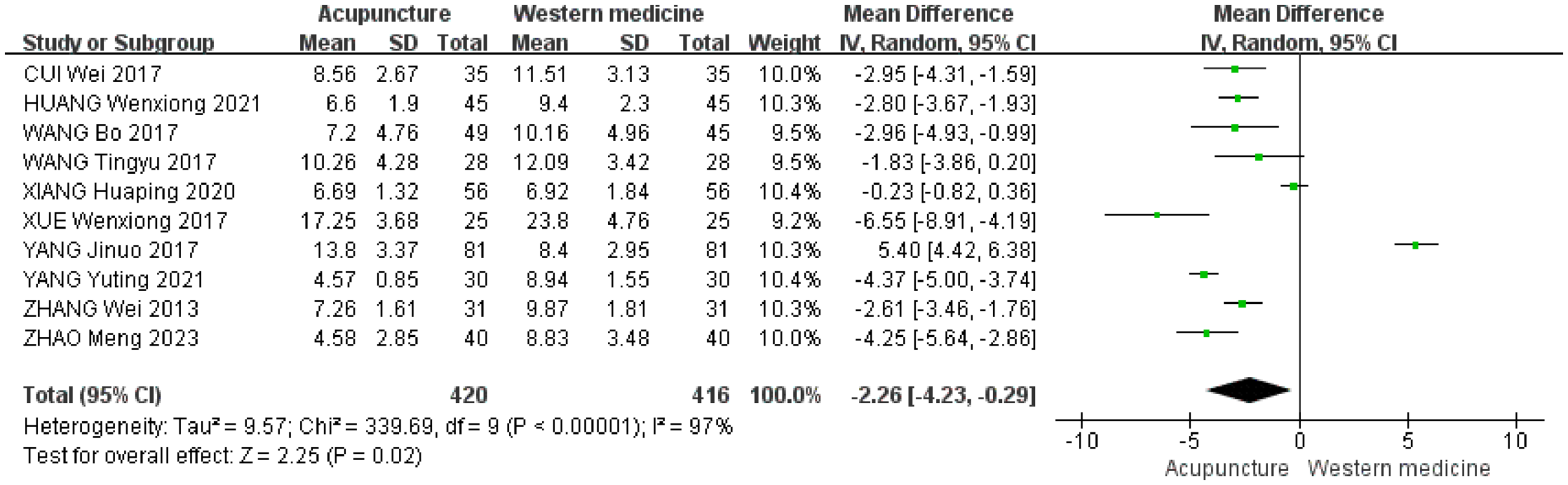
Ten studies on a forest plot (acupuncture vs. Western medicine).

This study performed a meta-analysis to assess the effects of acupuncture compared to conventional Western medicine on improving PSQI scores. A total of 10 studies were included, and the results from the random-effects model indicated that acupuncture significantly outperformed the drug control group in reducing PSQI scores (i.e., improving sleep quality), with a statistically significant difference [MD = −2.26, 95% CI (−4.23 to −0.29), *p* < 0.00001] (19–26, 29, 30). However, there was a high degree of heterogeneity between the studies (I^2^ = 97%), suggesting the presence of additional significant sources of heterogeneity beyond sampling errors (Figure 6).

**Figure 7:**
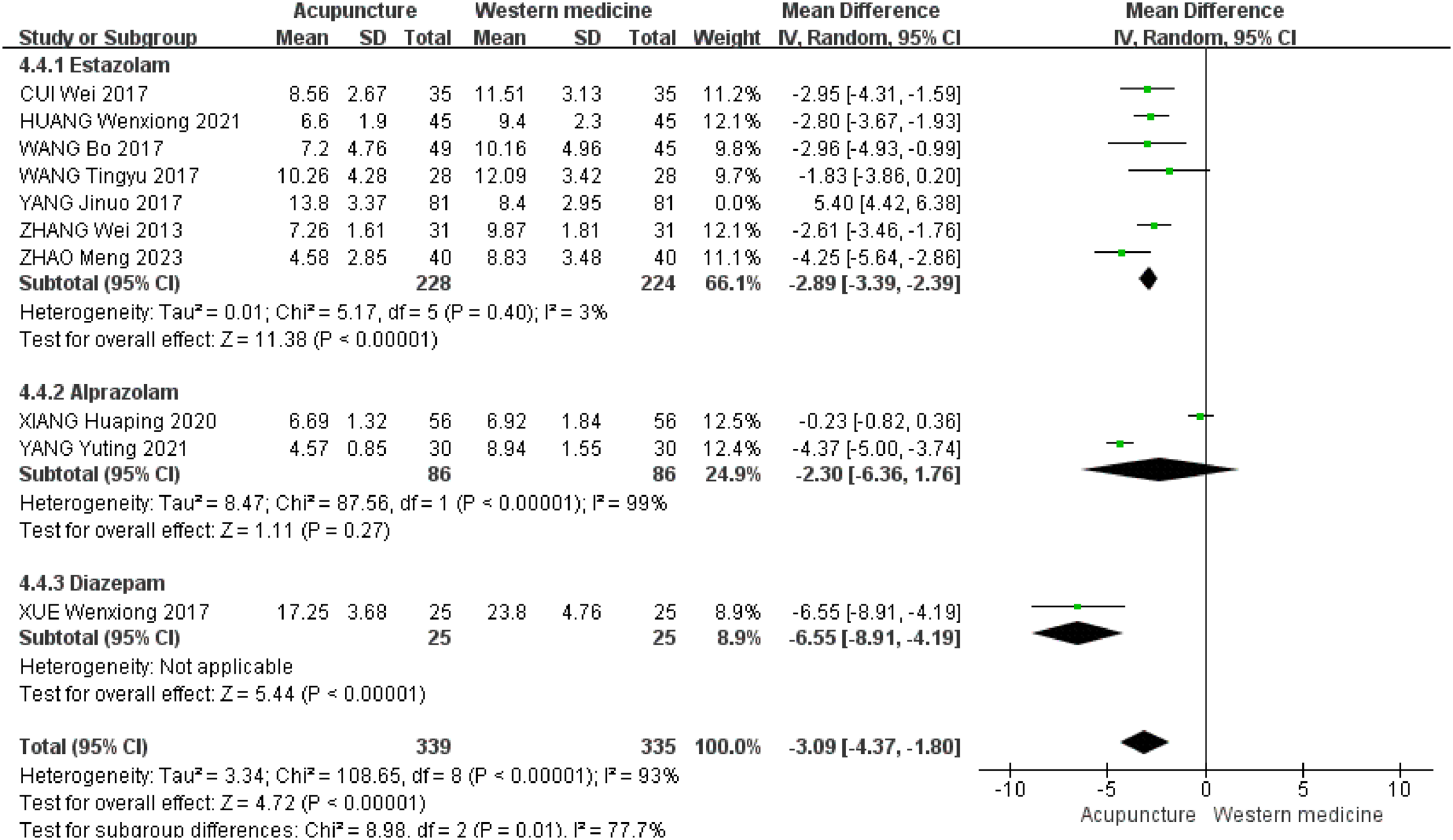
Subgroup analysis chart of acupuncture vs. different Western medicine types.

To further investigate the sources of heterogeneity, we conducted a subgroup analysis based on the types of Western medicines used in the control group. The results of the subgroup difference test were statistically significant (*χ*^2^ = 8.98, df = 2, *p* = 0.01, I^2^ = 77.7%), indicating that the type of Western medicine in the control group is a key factor influencing the overall heterogeneity. Subgroup analyses showed that seven studies used eszopiclone as a control treatment [MD = −2.89, 95% CI (−3.39 to −2.39), *p* < 0.00001] (19–21, 23, 25, 26, 30). Two studies used alprazolam as a control treatment [MD = −2.30, 95% CI (−6.36 to −1.76), *p* = 0.27] (22, 24). One study used diazepam as a control treatment [MD = −6.55, 95% CI (−8.91 to −4.19), *p* < 0.00001] (29) (Figure 7).

**Comparison of acupuncture and sham acupuncture.**

**Figure 8:**
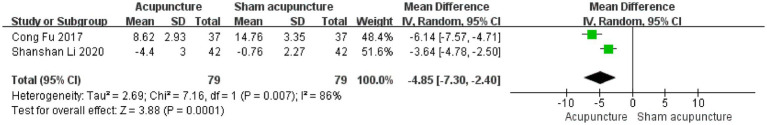
Acupuncture vs. Sham acupuncture forest plot.

Two studies assessed the specific efficacy of acupuncture by including a sham acupuncture control group (27, 28). The combined analysis revealed that the reduction in PSQI scores in the acupuncture group was significantly greater than in the sham acupuncture group, with a statistically significant difference [MD = −4.85, 95% CI (−7.30 to −2.40), *p* = 0.0001]. This result suggests that the efficacy of acupuncture in improving sleep is not solely attributable to the placebo effect, but that the specific acupuncture treatment itself has a significant positive impact. However, it is important to note the high heterogeneity between the results of these two studies (I^2^ = 86%), indicating that the combined effect size should be interpreted with caution (Figure 8).

### Secondary outcome indicators

#### LH

**Figure 9:**
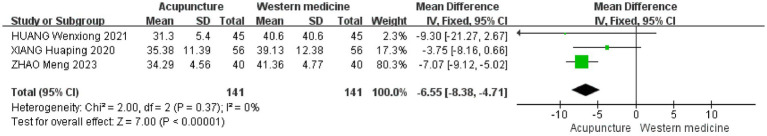
LH forest plot.

Only 3 studies evaluated LH levels (20, 22, 26). The forest plot indicated that acupuncture demonstrated greater efficacy than conventional Western medicine [MD = −6.55, 95% CI (−8.38 to −4.71), *p* < 0.00001] (Figure 9).

#### E2

**Figure 10:**
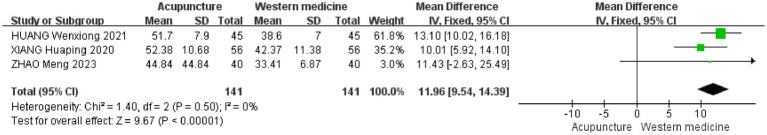
E2 forest plot.

Three studies evaluated E2 levels (20, 22, 26). The forest plot indicated that acupuncture demonstrated greater efficacy than conventional Western medicine mean difference [MD = 11.96, 95% CI (9.54 to 14.39), *p* < 0.00001] (Figure 10).

#### FSH

**Figure 11:**
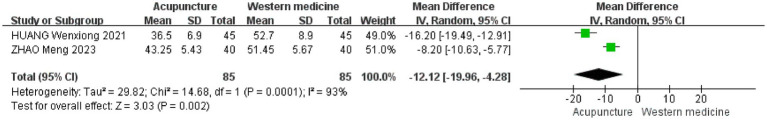
FSH forest plot.

Two studies evaluated FSH levels (20, 26). The forest plot indicated that acupuncture resulted in a significant reduction in FSH levels compared with the control group [MD = −12.12, 95% CI (−19.96 to −4.28), *p* = 0.002] (Figure 11).

#### ISI

**Figure 12:**
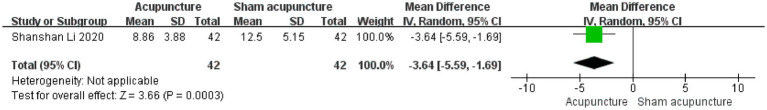
ISI forest plot.

Only 1 study recorded ISI levels, and the forest plot showed that the study was statistically significant [MD = −3.64, 95% CI (−5.59 to −1.69), *p* = 0.0003] (28) (Figure 12).

### Adverse events

The safety results and reporting quality summary of all included studies are presented in Table 2. After a systematic evaluation, only 4 (26–28, 30) of the 12 studies (19–30) provided quantitative adverse event (AE) data suitable for meta-analysis. In comparison, the remaining 8 studies did not explicitly report any safety-related data.

**Table 2:** Summary of safety outcomes and reporting quality in the included studies.

Study number	References	Withdrawal due to adverse events (trial group)	Withdrawal due to adverse events (control group)	Adverse events reported (trial group)	Adverse events reported (control group)	Comments
NO.1	Wei et al. (25)	Not reported	1 case	Not reported	Not reported	Although withdrawal cases were reported, the reasons were not explicitly attributed to adverse events. Additionally, the method of randomization was not described in detail.
NO.2	Bo et al. (30)	Not reported	Not reported	Dizziness, headache, nausea, anorexia, and fatigue (all ungraded) in 3 cases	Dizziness, headache, nausea, anorexia, and fatigue (all ungraded) in 38 cases	The report documented adverse event symptoms and the number of affected individuals. However, no standardized severity grading system was applied. The incidence rates differed significantly between the two groups, though the reasons for this disparity remain unclear.
NO.3	Yang (24)	Not reported	Not reported	Not reported	Not reported	No safety data were reported. The randomization method was not described in detail.
NO.4	Wei et al. (19)	Not reported	Not reported	Not reported	Not reported	No safety data were reported. The randomization method was not described in detail.
NO.5	Jinuo et al. (23)	Not reported	Not reported	Not reported	Not reported	No safety data were reported. The randomization method was not described in detail.
NO.6	Xue (29)	Not reported	Not reported	Not reported	Not reported	No safety data were reported. The randomization method was not described in detail.
NO.7	Xiang (22)	Not reported	Not reported	Not reported	Not reported	No safety data were reported. The randomization method was not described in detail.
NO.8	Zhao et al. (26)	Not reported	Not reported	0	0	No adverse events were reported (0 cases).
NO.9	Wang (2017) (32)	Not reported	Not reported	Not reported	Not reported	No safety data were reported. The randomization method was not described in detail.
NO.10	Wenxiong et al. (20)	Not reported	Not reported	Not reported	Not reported	No safety data were reported. The randomization method was not described in detail.
NO.11	Li et al. (28)	Not reported	Not reported	Bleeding (CTCAE grade 1), pain (CTCAE grade 1)-2 cases	Pain (CTCAE grade 1)-1 case	Provides AE data for analysis.
NO.12	Fu et al. (27)	1 case	1 case	0	Insomnia worsened-4 cases	Cases of withdrawal were reported, but the relationship between withdrawal and adverse events was not explicitly established. Additionally, the number of cases in the control group experiencing worsening insomnia (4 cases) was reported, but no severity grading was performed.

(1) CTCAE, Common Terminology Criteria for Adverse Events, version 5.0. Grades: 1 (mild), 2 (moderate), 3 (severe), 4 (life-threatening), 5 (death). (2) “Not reported” indicates that the item was not explicitly mentioned in the full text, tables, or figures of the study. (3) Given the widespread lack of safety data reporting in primary studies, this table provides a transparent overview of the reporting status of the current evidence base.

For the studies that reported AEs: one study (25), that reported one withdrawal in the control group, but the reason for withdrawal was not specified; one study (30) found that the incidence of ungraded AEs (including dizziness, headache, nausea, anorexia, and fatigue) in the control group (38 cases) was significantly higher than in the acupuncture group (three cases); one study (26) recorded no adverse events occurred in either group during the trial period (0 cases); one study (27) provided detailed data on CTCAE standard classification, with two cases of grade 1 bleeding and pain in the acupuncture group, and one case of grade 1 pain in the control group; one study (28) reported one withdrawal in both the trial and control groups, but the reasons for withdrawal were not specified. Additionally, four ungraded cases of aggravated insomnia were reported in the control group. The general inadequacy and irregularity in the reporting of safety data remain key limitations of the current available evidence.

**Figure 13:**
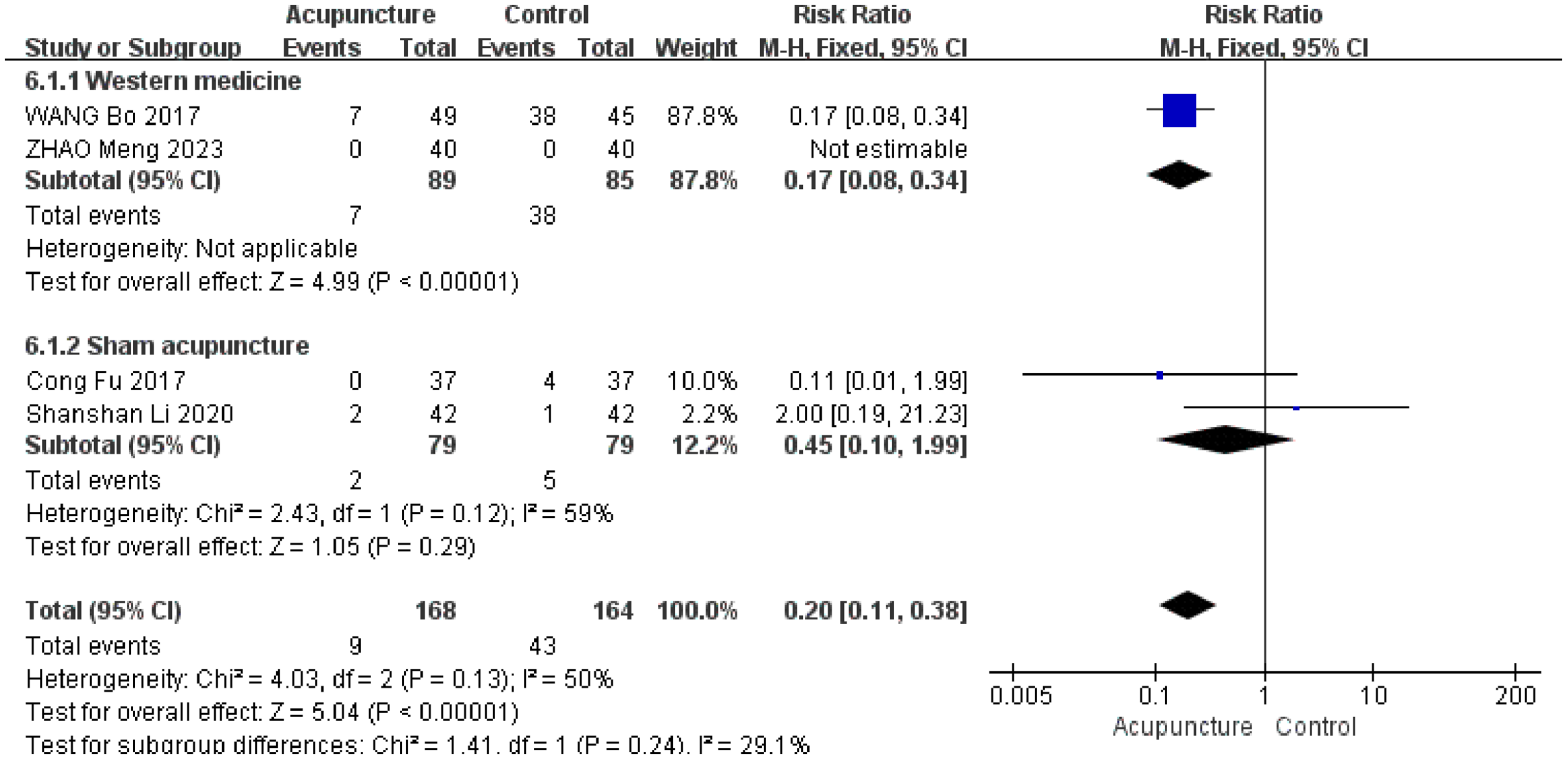
Adverse event forest plot.

We performed a subgroup meta-analysis based on the intervention measures of the control group. (1) Acupuncture vs. Western medicine: two studies (26, 30) were included. Adverse events in the Western medicine group (Estazolam) primarily included dizziness, headache, fatigue, nausea, and anorexia. In contrast, adverse events in the acupuncture group were limited to mild pain and bleeding at the acupuncture site. The meta-analysis results indicated that the incidence of adverse events in the acupuncture group was significantly lower than that in the Western medicine group [RR = 0.17, 95% CI (0.08 to 0.34), *p* < 0.00001] (Figure 13). (2) Acupuncture vs. Sham acupuncture: two studies were included. In the sham acupuncture group, one study reported mild acupuncture pain, while another reported four cases of severe insomnia. The acupuncture group reported mild adverse events, primarily pain and bleeding at the acupuncture site. The combined analysis revealed no statistically significant difference in the incidence of adverse events between the two groups [RR = 0.45, 95% CI (0.10 to 1.99), *p* = 0.29] (Figure 13). Moderate heterogeneity was observed in this subgroup (I^2^ = 59%). (3) The overall effect size from the combined studies showed no statistically significant difference between the two groups [RR = 0.20, 95% CI (0.11 to 0.38), *p* = 0.13], with moderate heterogeneity (I^2^ = 50%). No statistically significant difference was observed between subgroups (*p* = 0.24) (Figure 13). These results suggest that acupuncture is associated with fewer adverse events compared to Western medicine, but no significant difference was observed when compared with sham acupuncture. However, due to the small number of studies and limited sample size, this conclusion requires further verification through additional high-quality studies.

### Sensitivity analysis

#### Sensitivity analysis based on methodological quality

**Figure 14:**
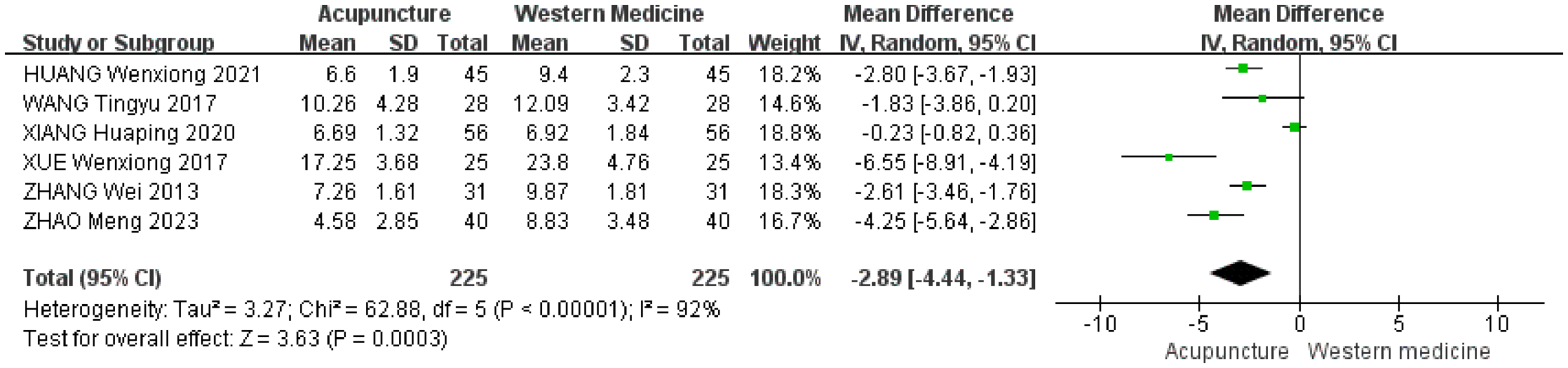
Sensitivity analysis forest plot.

A leave-one-out sensitivity analysis was conducted on the ten included drug-controlled studies to assess the impact of each study on the pooled results. We found that study (23) had a disproportionate influence on heterogeneity and the overall effect size. After data verification, this study was excluded from the analysis. Furthermore, due to the limited number of included studies, we adopted conservative and stringent criteria to define “low-quality studies,” specifically those that met both of the following conditions: (1) unclear description of randomization methods (i.e., only stating “random” without specifying the exact method), and (2) no blinding reported. We believe these criteria effectively capture key methodological flaws that could introduce bias. Based on these standards, we excluded a total of four studies. We then re-conducted the meta-analysis using a random-effects model for the remaining six studies. The sensitivity analysis results indicated a combined effect size of [MD = −2.89, 95% CI (−4.44 to −1.33), *p* = 0.0003] (Figure 14).

When compared to the results of the primary analysis (based on 10 studies) [MD = −2.26, 95% CI (−4.23 to −0.29), *p* < 0.00001] (Figure 6), we found the following: (1) the effect direction was entirely consistent, with both analyses showing a significant reduction in PSQI scores in the acupuncture group (both negative MDs); (2) statistically significant results, as both *p*-values were well below 0.05, confirming the strength of the findings; (3) changes in the effect size and their implications: The point estimate shifted from −2.26 to −2.89, suggesting that excluding studies with lower methodological quality revealed a greater efficacy advantage of acupuncture over Western medicine. The confidence intervals also reflected a reduction in heterogeneity (from I^2^ = 97 to 92%). Although the intervals widened, they still overlapped with those of the primary analysis and remained entirely to the left of the null effect line.

These findings support the robustness of our core conclusion that acupuncture is superior to Western medicine in improving PSQI scores. Notably, after excluding studies with a potential high risk of bias, the point estimate increased, further enhancing the reliability of our conclusion.

#### Sensitivity analysis on intervention heterogeneity (auricular acupuncture)

In addition to assessing methodological quality, we evaluated the potential impact of heterogeneity in acupuncture techniques on the pooled results. As previously noted, the “acupuncture” intervention group predominantly consisted of studies using conventional acupuncture (9 studies) (19–26, 29), with only one study (30) employing auricular acupuncture. To determine whether this single study influenced our conclusions, we conducted a sensitivity analysis by excluding the study (30). The results indicated that the change in effect size was minimal (before exclusion: SMD = −0.94, 95% CI: −1.73 to −0.14; after exclusion: SMD = −0.98, 95% CI: −1.89 to −0.07). Both statistical significance and the direction of the conclusion remained consistent (detailed in Supplementary Figure S6). These findings suggest that the overall conclusion regarding the efficacy of acupuncture, which primarily reflects the effects of conventional acupuncture, is robust despite the inclusion of this technically distinct intervention.

## Discussion

### Summary of findings

We included 12 RCTs for analysis, involving 994 patients, all of which were conducted in China. Current evidence of low to moderate quality suggests that acupuncture may offer potential benefits and demonstrate good safety for PMI. However, these findings require further validation through large-scale, multicenter, double-blind RCTs using standardized protocols.

### Clinical significance of the findings

The results of this study demonstrate that acupuncture significantly reduced the PSQI score of insomnia patients by −3.09 points (95% CI: −4.37 to −1.80) compared to conventional drug treatment. Notably, this effect size exceeds the recognized threshold for the minimally clinically important difference (MCID) of 3 points (31). This finding not only confirms the statistical advantages of acupuncture but, more importantly, establishes its clinical relevance. This reduction may translate into tangible, personally perceived improvements, such as a significant shortening of sleep time, a reduction in nighttime awakenings, and improved energy upon waking, all of which are critical for enhancing quality of life.

Additionally, the conclusions of this study are based on comparisons with active drug controls, further strengthening the evidence for acupuncture as an effective treatment option for insomnia. Despite the observed high heterogeneity, sensitivity analysis supports the robustness of the core conclusions. All included studies used the total PSQI score as the primary outcome measure. Future research should focus on specific PSQI dimensions (e.g., sleep latency and sleep efficiency) and examine the long-term persistence of acupuncture’s effects to assess its sustained clinical value more comprehensively.

### Heterogeneity source analysis

The included studies demonstrated high heterogeneity, possibly due to factors such as acupoint selection, type of acupuncture, treatment duration, practitioner skill, and overall treatment protocols. All these factors may influence the heterogeneity of the study results. However, due to the limited number of studies included, we were unable to conduct an in-depth subgroup analysis to identify specific sources of heterogeneity.

The studies included in this meta-analysis exhibit considerable heterogeneity, which represents a significant limitation. Notably, subgroup analysis revealed that the type of drug in the control group (e.g., alprazolam, estazolam, and diazepam) was a significant source of this heterogeneity (subgroup difference test, *p* = 0.01). This finding indicates that the comparative efficacy of acupuncture versus Western medicine may vary depending on the specific drug used. Variations in the pharmacological properties of these drugs (e.g., half-life, sedative efficacy, and side effects) and their baseline efficacy likely contribute to this observed difference.

The high heterogeneity observed in this study must be interpreted in the context of significant clinical variations in the acupuncture interventions themselves. As detailed in Supplementary Table S2, key parameters, such as stimulation techniques and total therapeutic doses, differ substantially across the included studies. While some studies employed the “even supplementation and even reduction” technique, others utilized “supplementing” or “lifting-thrusting and twirling method,” and some studies did not report this critical detail not report. These differences in treatment principles and operational techniques represent a significant source of clinical heterogeneity. Additionally, the total number of treatments varied widely, ranging from 10 to 28 sessions, resulting in a nearly threefold difference in the therapeutic dose received by patients. Furthermore, many studies failed to report essential details such as needle specifications and needle insertion depth, making it difficult to evaluate the standardization and repeatability of the interventions. These variations highlight the heterogeneous nature of acupuncture’s effects, with its efficacy likely influenced by factors such as technique, therapeutic dose, and the practitioner’s skill level. This clinical heterogeneity provides a reasonable and primary explanation for the high heterogeneity observed in our meta-analysis. Future studies should adopt more standardized trial designs and larger sample sizes to confirm these findings and explore the influence of additional factors on treatment efficacy.

### Comparison with similar studies

Our meta-analysis, updated to December 2023, incorporates the latest research. To our knowledge, previous reviews have shown that acupuncture is superior to sham acupuncture or standard Western medications in treating PMI, and there have also been reviews demonstrating the efficacy of combining Chinese herbs and acupuncture for PMI treatment. However, prior systematic reviews have only evaluated one control measure, whereas this study is the first to compare acupuncture with different control measures for PMI comprehensively. Furthermore, this study incorporates more comprehensive outcome indicators, such as PSQI, FSH, LH, ISI, and adverse events, compared to previous systematic reviews, thereby enhancing its rigor and completeness (15, 16). Significantly, earlier research primarily focused on short-term sleep improvements, whereas our review observes that acupuncture can improve long-term sleep quality in PMI. Some studies have documented long-term follow-up, demonstrating the sustained efficacy of acupuncture in treating PMI (27, 28, 30).

We also summarized the frequency of use of acupoints and found that the most used acupoints for treating insomnia in PMI are: Baihui (DU20), Anmian, Shenmen (HT7), Sishencong (EX-HN1), Sanyinjiao (SP6), Zhongwan (RN12), and Guanyuan (RN4). The frequent use of these acupoints is based on the profound understanding of the pathogenesis of PMI in traditional Chinese medicine. Traditional Chinese medicine holds that the core of PMI lies in the deficiency of kidney essence, which leads to the dysfunction of internal organs and the imbalance of yin and yang, often presenting symptoms such as the disharmony between the heart and kidney or liver depression with blood deficiency. DU20, EX-HN1, and HT7 have the effect of calming the mind and stabilizing the spirit, as well as clearing the heart and relieving annoyance. SP6 and RN4 nourish the liver and kidneys and strengthen the spleen to promote blood production. RN12 regulates the middle jiao, and Anmian is an effective empirical acupoint. The combination of these acupoints works together to balance yin and yang and calm the mind and stabilize the spirit, thereby effectively alleviating insomnia symptoms during perimenopause. This reflects the therapeutic principle of traditional Chinese medicine in treating PMI, which is to “harmonize heart and kidney, soothe the liver and strengthen the spleen, nourish blood and calm the spirit, and address both the symptoms and root causes.”

This study systematically evaluated adverse events associated with insomnia treatment through acupuncture. The meta-analysis results indicate that the incidence of adverse events in acupuncture is significantly lower than in conventional Western medicine, with no significant difference compared to sham acupuncture. This finding holds important clinical significance. First, acupuncture demonstrates notable safety advantages over Western medicine. Adverse events reported in the Western medicine group, such as dizziness, headache, fatigue, and nausea, are common side effects of benzodiazepines, which often compromise patients’ medication adherence and quality of life. The results suggest that acupuncture offers a non-pharmacological treatment option for insomnia, particularly for patients who are sensitive to drug side effects, have contraindications for medication, or seek long-term safe treatment. Second, when compared to sham acupuncture, no statistically significant difference in adverse event incidence was observed between the two groups, with both groups reporting mild adverse events (e.g., pain and bleeding at the acupuncture site). This finding enhances our understanding of acupuncture and supports its safety as an intervention. Moreover, acupuncture’s efficacy exceeds that of sham acupuncture, supporting the notion that its therapeutic effects are not associated with increased safety risks. However, due to the limited number of studies included and the small sample size for adverse events, the results warrant further verification through large-sample, long-term follow-up studies. Therefore, we recommend acupuncture as a treatment for PMI.

### Strengths

This study is the first to comprehensively compare acupuncture with various control measures (including conventional Western medicine and sham acupuncture) in the context of PMI. It demonstrates not only the efficacy advantages of acupuncture over active controls (Western medicine) but also confirms that its efficacy surpasses the placebo effect, as shown by the sham acupuncture control. Furthermore, in addition to the primary outcome indicator (PSQI), this study also evaluated hormone levels (FSH, LH, and E2), the ISI, and adverse events, thereby providing more comprehensive evidence for the efficacy and safety of acupuncture in treating PMI. In the face of considerable heterogeneity, detailed subgroup analysis (by type of Western medicine), sensitivity analysis (excluding low-quality studies and ear acupuncture studies), and publication bias assessment were conducted, demonstrating the robustness of the core conclusions. All included trials consistently employed “manual acupuncture” as the primary intervention, minimizing heterogeneity caused by different intervention methods (such as mixed hand needles and electroacupuncture), thereby ensuring the reliability and purity of the conclusions regarding the efficacy of “hand needles.” Additionally, this study highlighted that the reduction in PSQI score in the acupuncture group (−3.09) exceeded the minimum clinically important difference (MCID = 3), confirming that the improvement is not only statistically significant but also of clinical relevance.

## Limitations and future directions

Despite the strengths of this review, several limitations must be acknowledged when interpreting the findings. (1) Regarding the breadth and depth of evidence: The limited number of studies included in this review, primarily sourced from China, may restrict the generalizability of the results. Additionally, few studies have examined hormone levels and ISI, and these findings require further validation. Therefore, future research should incorporate a broader range of literature and expand the investigation of specific outcome indicators to establish a more comprehensive and reliable evidence base. (2) About covariate analysis: This review does not explore the potential effects of important covariates, such as age or baseline insomnia severity (e.g., PSQI scores), on the efficacy of acupuncture therapy. Despite our efforts to conduct relevant subgroup analyses, we were unable to perform this analysis because none of the original studies reported outcome data stratified by these factors. As a result, we cannot determine whether acupuncture therapy is more effective in specific patient groups, which limits the ability to provide personalized treatment recommendations. We strongly recommend that future clinical trials pre-plan subgroup analyses and transparently report the associated data. (3) Due to the limited number of studies included, we were unable to use the full Cochrane Risk of Bias 2 (RoB 2) tool for sensitivity analysis. Instead, we adopted simplified criteria based on key methodological elements, such as randomization and blinding, to define “low-quality research.” These criteria were designed to identify studies with a high risk of bias conservatively, and the results support the robustness of the main conclusions. (4) One of the main limitations of this review is the inability to conduct subgroup analyses based on acupuncture techniques to explore potential differences in treatment effects across methods. The study does not address electroacupuncture and includes only one study on auricular acupuncture. Therefore, the findings are primarily applicable to conventional acupuncture and cannot be directly extended to electroacupuncture or auricular acupuncture. Future research should directly compare the efficacy of manual acupuncture, electroacupuncture, and other acupuncture techniques to provide evidence that supports the clinical selection of the most appropriate treatment regimen. (5) This study has an important limitation concerning the methodological quality of the included trials. The majority of studies demonstrated a high risk of bias, primarily due to the widespread lack of blinding for both participants and practitioners. Given that the primary outcome measure (PSQI) is subjective, this issue is likely to introduce significant performance and detection biases. Specifically, patients in the treatment group who were aware of their treatment allocation may have been more inclined to report positive outcomes due to expectation effects, leading to an overestimation of the efficacy of acupuncture compared to non-blinded controls. However, it is worth noting that acupuncture still demonstrated significant efficacy when compared to a sham acupuncture control group, where blinding was implemented. This suggests that acupuncture’s therapeutic effect extends beyond a placebo response. Furthermore, the high heterogeneity observed in the meta-analysis may partly result from variations in the risk of bias among the included studies. While the results suggest that acupuncture is effective for perimenopausal insomnia (PMI), the effect sizes derived from comparisons with non-blinded control groups should be interpreted with caution due to these limitations. To validate these findings, future high-quality RCTs with rigorous blinding procedures, objective outcome measures, and comprehensive reporting standards are urgently needed. (6) This study highlights that over one-third of the included studies did not provide detailed information on the methods for generating random sequences. Most of the studies failed to specify the allocation concealment procedures clearly. These methodological shortcomings may lead to selection bias, potentially inflating the estimated intervention effect. To enhance the quality and reliability of future research, we strongly recommend the use of robust randomization and allocation concealment techniques, such as central randomization systems or sequentially numbered, sealed envelopes. At the same time, studies should adhere to the Consolidated Standards of Reporting Trials (CONSORT) Statement, particularly its updated standards regarding randomization, and follow the STRICTA guidelines for acupuncture trials to ensure standardized reporting, thereby improving research transparency and the credibility of findings. (7) This review was limited by the inconsistent and often incomplete reporting of acupuncture parameters (e.g., needle type and insertion depth) in the original studies. Although we comprehensively extracted all available details (Supplementary Table S2) and conducted qualitative assessments based on the STRICTA guidelines, the small number of studies and the lack of key information prevented us from quantifying the contribution of specific techniques to the outcomes, which may affect the interpretation of technique-specific effects. Therefore, we strongly recommend that future clinical studies rigorously follow the STRICTA reporting guidelines to improve the quality, comparability, and clinical applicability of the evidence. (8) Testing of major outcome indicators suggests potential publication bias. The results of Egger’s test were highly significant, indicating that our findings may be influenced by the absence of unpublished small negative studies, which could lead to an overestimation of the true effect of acupuncture in treating insomnia. Although we attempted to correct this bias using the trim-and-fill method, the results were not reported because we failed to obtain stable estimates. Therefore, readers should interpret the conclusions of this study with caution, recognizing the possibility that the combined effect size may be overestimated. Further large-scale, high-quality studies are needed to confirm these results in the future. (9) We explicitly state that significant clinical heterogeneity exists between the interventions and control measures in the studies, which precludes an effective GRADEpro evaluation. We suggest that future research should focus on more standardized interventions and control measures to enable high-quality meta-analyses and reliable GRADEpro evaluations.

## Conclusion

Current evidence of low to moderate quality suggests that acupuncture may offer potential benefits and demonstrate good safety for PMI. However, these findings require further validation through large-scale, multicenter, double-blind RCTs using standardized protocols.

## Data Availability

The original contributions presented in the study are included in the article/Supplementary material, further inquiries can be directed to the corresponding authors.
