# Supplementary figures and images for: Acupuncture for perimenopausal insomnia: a systematic review and meta-analysis
Source: Front Med (Lausanne). 2025 Oct 13;12:1673994. doi: 10.3389/fmed.2025.1673994 (PMC12554702; doi:10.3389/fmed.2025.1673994)

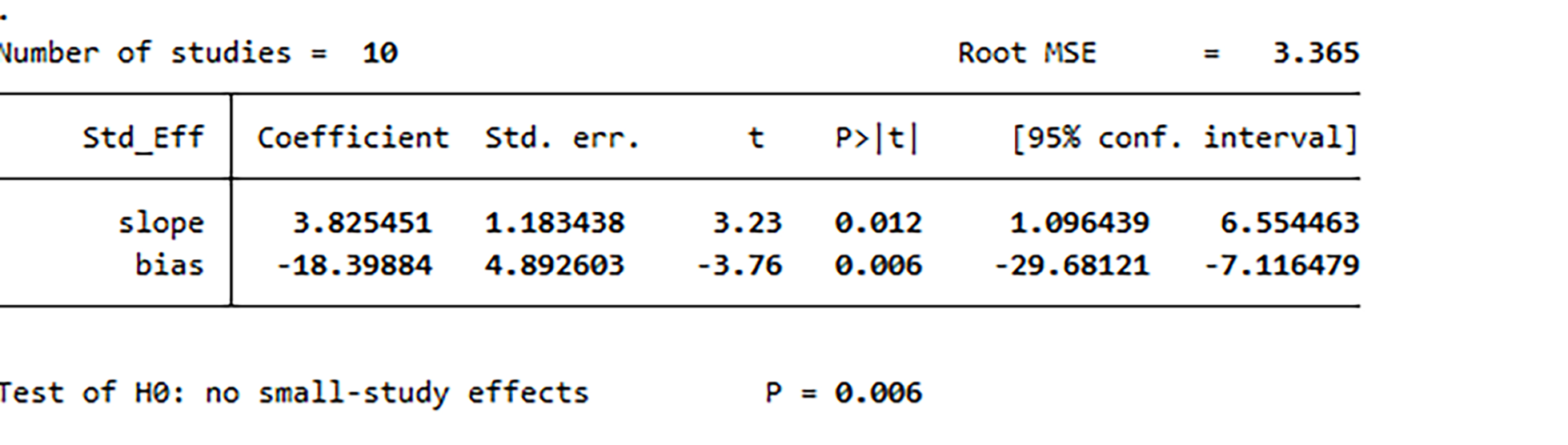

Supplement: Supplementary file 1 [file Image_1.TIF]

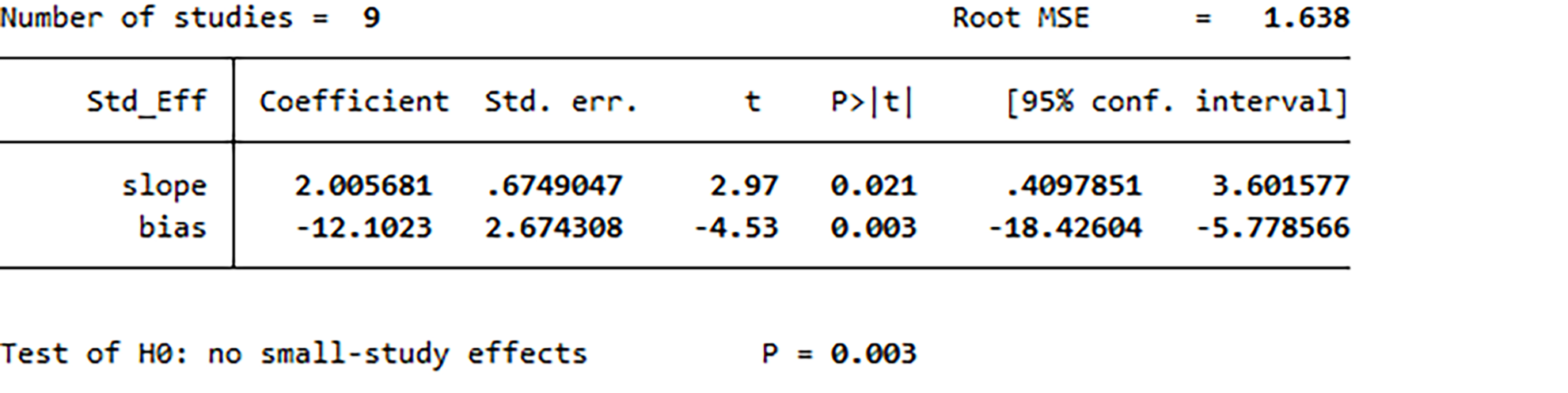

Supplement: Supplementary file 2 [file Image_2.TIF]

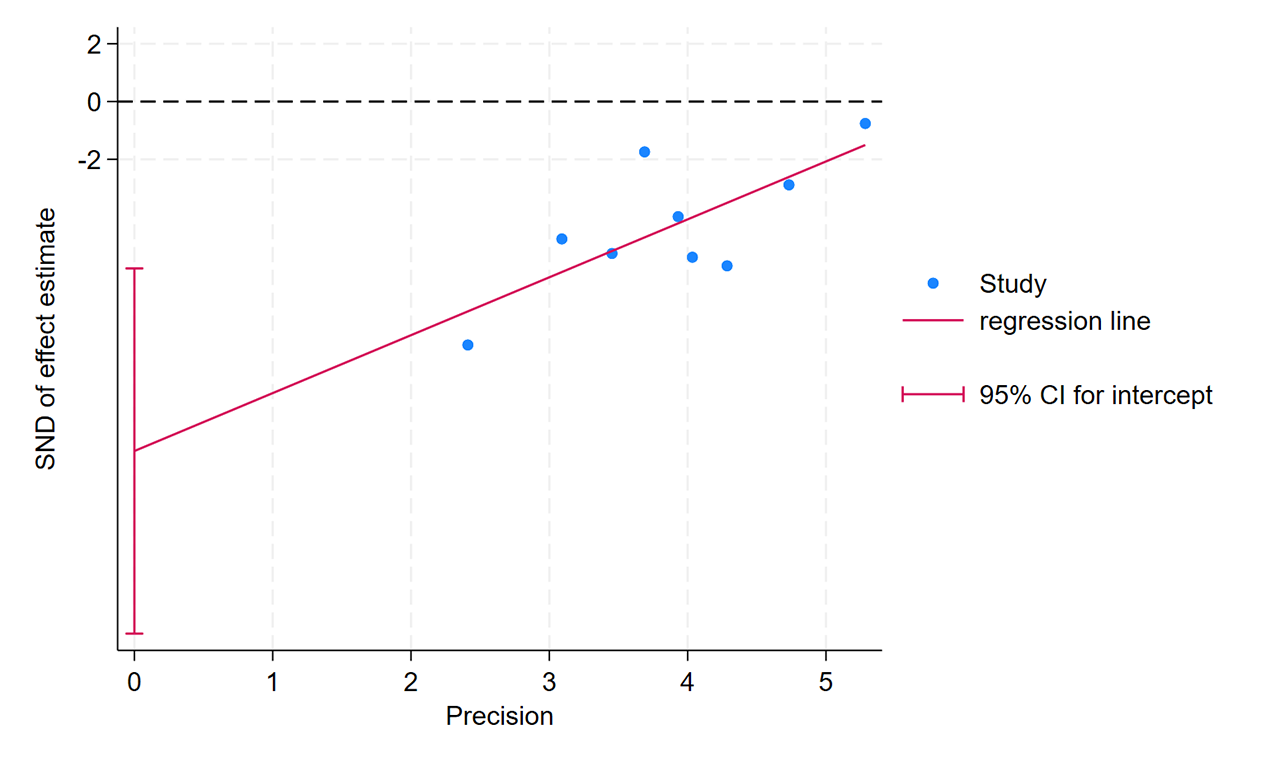

Supplement: Supplementary file 5 [file Image_5.TIF]
