# Supplementary material for: Acupuncture for perimenopausal insomnia: a systematic review and meta-analysis
Source: Front Med (Lausanne). 2025 Oct 13;12:1673994. doi: 10.3389/fmed.2025.1673994 (PMC12554702; doi:10.3389/fmed.2025.1673994)
Supplement: Supplementary file 7 [file Table_2.DOCX]

The Supplementary Material 2: Characteristics of interventions included in studies (based on STRICTA guidelines)

| **Number** | **Author**  **(First Author)** | **Year** | **Group** | **Intervention measures** | **Needle Specifications** | **Needle insertion depth** | **Needle retention time** | **Stimulation technique** | **Treatment frequency** | **Total Course of Treatment** |
| --- | --- | --- | --- | --- | --- | --- | --- | --- | --- | --- |
| NO.1 | Zhang Wei | 2013 | Treatment group | Acupuncture | NR | NR | 30 minutes | Neutral tonification and neutral purgation | Once daily, five times a week | 4 weeks |
|  |  |  | Control group | Medicines | N/A | N/A | N/A | N/A | Once every night before bedtime | 4 weeks |
| NO.2 | Wang Bo | 2017 | Treatment group | Auricular Acupuncture Points | NR | NR | Press for 30 seconds each time. | NR | Press 3 times daily | 6 weeks |
|  |  |  | Control group | Medicines | N/A | N/A | N/A | N/A | Once every night before bedtime | 6 weeks |
| NO.3 | Yang Yuting | 2021 | Treatment group | Acupuncture | NR | NR | 30 minutes | NR | 3 times a week | 4 weeks |
|  |  |  | Control group | Medicines | N/A | N/A | N/A | N/A | Once every night before bedtime | 4 weeks |
| NO.4 | Cui Wei | 2017 | Treatment group | Acupuncture | NR | NR | 30 minutes | NR | Every other day | 4 weeks |
|  |  |  | Control group | Medicines | N/A | N/A | N/A | N/A | Once every night before bedtime | 4 weeks |
| NO.5 | Yang Jinuo | 2017 | Treatment group | Acupuncture | NR | NR | NR | NR | NR | 12 weeks |
|  |  |  | Control group | Medicines | N/A | N/A | N/A | N/A | NR | 12 weeks |
| NO.6 | Xue Wenxiong | 2017 | Treatment group | Acupuncture | NR | 0.3 to 1.5 inches | 30 minutes | Method of supplementation | Once daily | 2 weeks |
|  |  |  | Control group | Medicines | N/A | N/A | N/A | N/A | Once every night before bedtime | 2 weeks |
| NO.7 | Xiang Huaping | 2020 | Treatment group | Acupuncture | 0.5 * 0.5 mm | 10-25 mm | 30 minutes | Neutral tonification and neutral purgation | Every other day | 9 weeks |
|  |  |  | Control group | Medicines | N/A | N/A | N/A | N/A | Once every night before bedtime | 9 weeks |
| NO.8 | Zhao Meng | 2023 | Treatment group | Acupuncture | Huatuo Brand 0.3 * 25 mm | 10-15 mm | 30 minutes | Neutral tonification and neutral purgation | Once daily | 4 weeks |
|  |  |  | Control group | Medicines | N/A | N/A | N/A | N/A | Once every night before bedtime | 4 weeks |
| NO.9 | Wang Tingyu | 2017 | Treatment group | Acupuncture | NR | NR | 30 minutes | Neutral tonification and neutral purgation | Once daily | 4 weeks |
|  |  |  | Control group | Medicines | N/A | N/A | N/A | N/A | Once every night before bedtime | 4 weeks |
| NO.10 | Huang Wenxiong | 2021 | Treatment group | Acupuncture | 0.3 * 40 mm | 1-1.5 cm | 30 minutes | Neutral tonification and neutral purgation | Once daily | 8 weeks |
|  |  |  | Control group | Medicines | N/A | N/A | N/A | N/A | Once every night before bedtime | 8 weeks |
| NO.11 | Shanshan Li | 2020 | Acupuncture Group | Acupuncture | Jiajian brand sterile needles, 0.25 * 40 mm and 0.30 * 40 mm | 10–30 mm | 30 minutes | Pull, insert, twist | Every other day | 8 weeks |
|  |  |  | Sham acupuncture group | Streitberger Comfort Needle | Streitberger placebo needle | N/A | 30 minutes |  | Every other day | 8 weeks |
| NO.12 | Cong Fu | 2017 | Acupuncture Group | Acupuncture | 0.25 * 40 mm | 10–30 mm | 20 minutes | Neutral tonification and neutral purgation | Every other day | 10 times |
|  |  |  | Sham acupuncture group | Streitberger Comfort Needle | Streitberger Comfort Needle | Non-invasive, does not penetrate the skin | 20 minutes | Comfort needle device simulates the sensation of acupuncture | Every other day | 10 times |

(1) This form is developed based on the STRICTA (Standards for Reporting Clinical Trials of Acupuncture Interventions) guidelines for systematically extracting and reporting details of acupuncture interventions.

(2) Not applicable (N/A); for unreported parameters, uniformly label as “Not reported (NR)”.

(3) Streitberger sham needle: Non-invasive, with a blunted needle tip that retracts upon skin contact to simulate the sensation of needling.

(4) Sham needle device simulating needling sensation: A blunt-tipped cannula retracts to produce a tactile sensation resembling insertion, without actual needle penetration.
