# Supplementary material for: Acupuncture for perimenopausal insomnia: a systematic review and meta-analysis
Source: Front Med (Lausanne). 2025 Oct 13;12:1673994. doi: 10.3389/fmed.2025.1673994 (PMC12554702; doi:10.3389/fmed.2025.1673994)
Supplement: Supplementary file 8 [file Table_3.DOCX]

**Search strategies of this study**

**Search strategies:**

(Search from database establishment to December 31, 2023)

**Pubmed**

| **No.** | **Query** |
| --- | --- |
| #1 | "Sleep Initiation and Maintenance Disorders"[MeSH Terms] |
| #2 | (((((((((((((((((((((((((Disorders of Initiating[Title/Abstract] OR Maintaining Sleep[Title/Abstract]) OR (Early Awakening[Title/Abstract])) OR (Awakening, Early[Title/Abstract])) OR (Awakening, Early[Title/Abstract])) OR (Insomnia, Nonorganic[Title/Abstract])) OR (Insomnia, Nonorganic[Title/Abstract])) OR (Insomnia, Primary[Title/Abstract])) OR (Transient Insomnia[Title/Abstract])) OR (Insomnia, Transient[Title/Abstract])) OR (Rebound Insomnia[Title/Abstract])) OR (Insomnia, Rebound[Title/Abstract])) OR (Secondary Insomnia[Title/Abstract])) OR (Insomnia, Secondary[Title/Abstract])) OR (Sleep Initiation Dysfunction[Title/Abstract])) OR (Dysfunction, Sleep Initiation[Title/Abstract])) OR (Dysfunction, Sleep Initiation[Title/Abstract])) OR (Sleep Initiation Dysfunctions[Title/Abstract])) OR (Sleeplessness[Title/Abstract])) OR (Insomnia Disorder[Title/Abstract])) OR (Insomnia Disorders[Title/Abstract])) OR (Insomnia[Title/Abstract])) OR (Insomnias[Title/Abstract])) OR (Chronic Insomnia[Title/Abstract])) OR (Insomnia, Chronic[Title/Abstract])) OR (Psychophysiological Insomnia[Title/Abstract])) OR (Insomnia, Psychophysiological[Title/Abstract]) |
| #3 | #1 OR #2 |
| #4 | Perimenopause"[MeSH Terms] |
| #5 | "Climacteric"[Title/Abstract] OR "Menopause"[Title/Abstract] |
| #6 | #4 OR #5 |
| #7 | Acupuncture"[MeSH Terms] |
| #8 | (((((((((((((((((Acupoint[Title/Abstract]) OR (Meridian[Title/Abstract])) OR (Electroacupuncture[Title/Abstract])) OR (Transcutaneous electrical nerve stimulation[Title/Abstract])) OR (Acupoint catgut embedding[Title/Abstract])) OR (Acupressure[Title/Abstract])) OR (Cupping jar[Title/Abstract])) OR (Moxibustion[Title/Abstract])) OR (Auricular points[Title/Abstract])) OR (Abdominal acupuncture points[Title/Abstract])) OR (Scalp acupuncture points[Title/Abstract])) OR (Laser[Title/Abstract])) OR (Magnets[Title/Abstract])) OR (Bleeding[Title/Abstract])) OR (Acupoint injection[Title/Abstract])) OR (Fire needle[Title/Abstract])) OR (Needle knife[Title/Abstract])) OR (Superficial needling[Title/Abstract]) |
| #9 | #8 OR #9 |
| #10 | "randomized controlled trial"[Publication Type] OR "randomized"[Title/Abstract] OR "placebo"[Title/Abstract] |
| #11 | #3 AND #6 AND #9 AND #10 |

**Embase**

| **No.** | **Query** |
| --- | --- |
| #1 | 'insomnia'/exp |
| #2 | 'maintenance disorders':ab,ti OR 'disorders of initiating and maintaining sleep':ab,ti OR 'dims (disorders of initiating and maintaining sleep)':ab,ti OR 'early awakening':ab,ti OR 'awakening, early':ab,ti OR 'nonorganic insomnia':ab,ti OR 'insomnia, nonorganic':ab,ti OR 'primary insomnia':ab,ti OR 'insomnia, primary':ab,ti OR 'transient insomnia':ab,ti OR 'insomnia, transient':ab,ti OR 'rebound insomnia':ab,ti OR 'insomnia, rebound':ab,ti OR 'secondary insomnia':ab,ti OR 'insomnia, secondary':ab,ti OR 'sleep initiation dysfunction':ab,ti OR 'dysfunction, sleep initiation':ab,ti OR 'dysfunctions, sleep initiation':ab,ti OR 'sleep initiation dysfunctions':ab,ti OR 'sleeplessness':ab,ti OR 'insomnia disorder':ab,ti OR 'insomnia disorders':ab,ti OR 'insomnia':ab,ti OR 'insomnias':ab,ti OR 'chronic insomnia':ab,ti OR 'insomnia, chronic':ab,ti OR 'psychophysiological insomnia':ab,ti OR 'insomnia, psychophysiological':ab,ti |
| #3 | 'climacterium'/exp OR 'climacterium' |
| #4 | 'perimenopausal':ab,ti OR 'menopause':ab,ti |
| #5 | #1 OR #2 |
| #6 | #3 OR #4 |
| #7 | 'acupuncture'/exp OR 'acupuncture' |
| #8 | acupuncture AND therapy |
| #9 | #7 OR #8 |
| #10 | 'randomized controlled trial':ab,ti OR 'randomized':ab,ti OR 'placebo':ab,ti |
| #11 | #5 AND #6 AND #9 AND #10 |

**Cochrane**

| **No.** | **Query** |
| --- | --- |
| #1 | MeSH descriptor: [Sleep Initiation and Maintenance Disorders] explode all trees |
| #2 | (Maintenance Disorders):ti,ab,kw OR (Disorders of Initiating and Maintaining Sleep):ti,ab,kw |
| #3 | (Disorders of Initiating and Maintaining Sleep):ti,ab,kw OR (Early Awakening):ti,ab,kw OR (Awakening, Early):ti,ab,kw OR (Nonorganic Insomnia):ti,ab,kw OR (Insomnia, Nonorganic):ti,ab,kw OR (Primary Insomnia):ti,ab,kw OR (Insomnia, Primary):ti,ab,kw OR (Transient Insomnia):ti,ab,kw OR (Insomnia, Transient):ti,ab,kw OR (Rebound Insomnia):ti,ab,kw OR (Insomnia, Rebound):ti,ab,kw OR (Secondary Insomnia):ti,ab,kw OR (Insomnia, Secondary):ti,ab,kw OR (Sleep Initiation Dysfunction):ti,ab,kw OR (Dysfunction, Sleep Initiation):ti,ab,kw OR (Dysfunctions, Sleep Initiation):ti,ab,kw OR (Sleep Initiation Dysfunctions):ti,ab,kw OR (Sleeplessness):ti,ab,kw OR (Insomnia Disorder):ti,ab,kw OR (Insomnia Disorders):ti,ab,kw OR (Insomnia):ti,ab,kw OR (Insomnias):ti,ab,kw OR (Chronic Insomnia):ti,ab,kw OR (Insomnia, Chronic):ti,ab,kw OR (Psychophysiological Insomnia):ti,ab,kw OR (Insomnia, Psychophysiological):ti,ab,kw |
| #4 | #1 OR #2 OR #3 |
| #5 | MeSH descriptor: [Perimenopause] explode all trees |
| #6 | (Climacteric ):ti,ab,kw OR (Menopause):ti,ab,kw OR (Menopause, Premature):ti,ab,kw OR (Postmenopause):ti,ab,kw |
| #7 | #5 OR #6 |
| #8 | #4 and #7 |
| #9 | MeSH descriptor: [Acupuncture] explode all trees |
| #10 | (Acupoint):ti,ab,kw OR ( Meridian):ti,ab,kw OR (Electroacupuncture):ti,ab,kw OR ( Transcutaneous electrical nerve stimulation):ti,ab,kw OR (Acupoint catgut embedding):ti,ab,kw OR (Acupressure):ti,ab,kw OR ( Cupping jar):ti,ab,kw OR (Moxibustion):ti,ab,kw OR (Auricular points):ti,ab,kw OR ( Abdominal acupuncture points):ti,ab,kw OR (Scalp acupuncture points):ti,ab,kw OR ( Laser):ti,ab,kw OR (Magnets):ti,ab,kw OR (Bleeding):ti,ab,kw OR ( Acupoint injection):ti,ab,kw OR (Fire needle):ti,ab,kw OR (Needle knife):ti,ab,kw OR (Superficial needling):ti,ab,kw |
| #11 | #9 OR #10 |
| #12 | #8 and #11 |

**Web of Science**

| **No.** | **Query** |
| --- | --- |
| #1 | insomnia (Topic) OR Sleep Initiation and Maintenance Disorders (Topic) OR （Disorders of Initiating and Maintaining Sleep or Disorders of Initiating and Maintaining Sleep or Early Awakening or Nonorganic Insomnia or Primary Insomnia or Transient Insomnia or Transient Insomnia or Secondary Insomnia Or Sleep Initiation Dysfunction or Sleep Initiation Dysfunctions or Sleeplessness or Insomnia Disorder or Insomnia Disorders or Insomnias or Chronic Insomnia or Psychophysiological Insomnia） (Topic) |
| #2 | Perimenopausal (Topic) OR Climacteric (Topic) OR Menopause (Topic) |
| #3 | #1 AND #2 |
| #4 | acupuncture (Topic) OR （Acupoint or Meridian or Electroacupuncture or Electroacupuncture or Transcutaneous electrical nerve stimulation or Acupoint catgut embedding or Acupressure or Cupping jar or Moxibustion or Auricular points or Abdominal acupuncture points or Scalp acupuncture points or Laser or Magnets or Bleeding or Acupoint injection or Fire needle or Needle knife or Superficial needling） (Topic) |
| #5 | #3 AND #4 |

**CNKI**

（主题=更年期失眠 OR 围绝经期失眠 OR 更年期睡眠障碍 OR 围绝经期睡眠障碍）AND （主题=针灸 OR 穴位 OR 经络 OR 电针 OR 经皮神经电刺激 OR 穴位埋线 OR 针压法 OR 火罐 OR 艾灸 OR 耳穴 OR 激光 OR 磁 OR 穴位注射 OR 放血 OR 火针 OR 针刀 OR 浮针) AND (摘要=随机 OR 随机对照试验 OR 临床对照试验 OR RCT)

**VIP**

（主题= 更年期失眠 OR 围绝经期失眠 OR 更年期睡眠障碍 OR 围绝经期睡眠障碍）and （主题= 针灸 OR 穴位 OR 经络 OR 电针 OR 经皮神经电刺激 OR 穴位埋线 OR 针压法 OR 火罐 OR 艾灸 OR 耳穴 OR 激光 OR 磁 OR 穴位注射 OR 放血 OR 火针 OR 针刀 OR 浮针）and（摘要= 随机 OR 随机对照试验 OR 临床对照试验 OR RCT）

**WF**

（主题=更年期失眠 OR 围绝经期失眠 OR 更年期睡眠障碍 OR 围绝经期睡眠障碍）AND （主题=针灸 OR 穴位 OR 经络 OR 电针 OR 经皮神经电刺激 OR 穴位埋线 OR 针压法 OR 火罐 OR 艾灸 OR 耳穴 OR 激光 OR 磁 OR 穴位注射 OR 放血 OR 火针 OR 针刀 OR 浮针) AND (摘要=随机 OR 随机对照试验 OR 临床对照试验 OR RCT)

**CBM**

| 序号 | 检索表达式 |
| --- | --- |
| 1 | 更年期失眠 |
| 2 | 围绝经期失眠 |
| 3 | "更年期睡眠障碍"[常用字段:智能] |
| 4 | "围绝经期更年期失眠"[常用字段:智能] |
| 5 | (#4) OR (#3) OR (#2) OR (#1) |
| 6 | "随机对照试验"[不加权:扩展] |
| 7 | "随机"[常用字段:智能] |
| 8 | "对照临床试验"[常用字段:智能] |
| 9 | "试验"[常用字段:智能] |
| 10 | (#9) OR (#8) OR (#7) OR (#6) |
| 11 | "针灸"[不加权:扩展] |
| 12 | "针刺疗法"[常用字段:智能] |
| 13 | "经络"[常用字段:智能] |
| 14 | "电针"[常用字段:智能] |
| 15 | "针灸穴位"[常用字段:智能] |
| 16 | "经皮神经电刺激"[常用字段:智能] |
| 18 | "穴位压"[常用字段:智能] |
| 19 | "艾灸"[常用字段:智能] |
| 20 | "耳穴"[常用字段:智能] |
| 21 | "腹部针灸"[常用字段:智能] |
| 22 | "头皮穴位"[常用字段:智能] |
| 23 | "穴位注射"[常用字段:智能] |
| 24 | "火针"[常用字段:智能] |
| 25 | "火针"[常用字段:智能] |
| 26 | "浅表针刺"[常用字段:智能] |
| 27 | "火罐"[常用字段:智能] |
| 28 | "激光"[常用字段:智能] |
| 29 | "磁铁"[常用字段:智能] |
| 30 | "出血"[常用字段:智能] |
| 31 | "穴位埋线"[常用字段:智能] |
| 32 | "浮刺"[常用字段:智能] |
| 33 | (#32) OR (#31) OR (#30) OR (#29) OR (#28) OR (#27) OR (#26) OR (#25) OR (#24) OR (#23) OR (#22) OR (#21) OR (#20) OR (#19) OR (#18) OR (#16) OR (#15) OR (#14) OR (#13) OR (#12) OR (#11) |
| 34 | (#33) AND (#10) AND (#5) |
